# The Influence of Heat Treatment on the Tensile Creep Life of the TC25 Titanium Alloy

**DOI:** 10.3390/ma17235821

**Published:** 2024-11-27

**Authors:** Ang Tian, Jianglong Ma, Zhiguo Liu, Guangming Kong, Jiacheng Geng, Xinyuan Huang, Guanliang Li

**Affiliations:** 1 School of Metallurgy, Northeastern University, Shenyang 110819, China; mjl15923484822@163.com (J.M.); gjc17863800508@163.com (J.G.); 20213625@stu.neu.edu.cn (X.H.); 2 Naval Aviation University Qingdao Campus, Qingdao 266041, China; qdnuaalzg@163.com (Z.L.); guangmink@163.com (G.K.); 3 Institute of Metal Research, Chinese Academy of Sciences, Shenyang 110016, China; glli22s@imr.ac.cn; 4 School of Materials Science and Engineering, University of Science and Technology of China, Shenyang 110016, China

**Keywords:** TC25 titanium alloy, heat treatment, creep behavior, creep fracture surface, creep mechanism

## Abstract

High-temperature titanium alloys are significant materials in the aerospace field, and their service life largely depends on creep aging. However, the creep behavior of the TC25 titanium alloy at high temperatures has not been reported. Here, the creep behavior of TC25 before and after heat treatment at 550 °C under different stresses was investigated. It was found that heat treatment significantly enhanced the creep resistance of the TC25 alloy. An increase in creep stress increased the steady-state creep rate and reduced creep life. The smooth αp/βtrans grain boundaries and refined αs improved creep resistance, and the creep mechanism changed from grain boundary sliding to dislocation climbing after heat treatment. This research provides theoretical data support for the application of the TC25 alloy at high temperatures.

## 1. Introduction

High-temperature titanium alloys are widely used in jet engines as fan blades, compressor blades, and disks in order to improve the thrust–weight ratio of the engine and the maneuverability of the aircraft due to their characteristics of low density and high specific strength [1,2,3,4,5]. Creep failure is an important factor affecting the application of high-temperature titanium alloys [5,6], and creep performance at high temperatures is related to the microstructure [7,8]. Creep behavior, as an important indicator of reliability in the application of high-temperature titanium alloys, is a research hotspot of high-temperature titanium alloys [1]. The microstructure of high-temperature titanium alloys can be effectively improved through heat treatment, thereby altering their mechanical properties [9,10,11]. Nie et al. obtained the lamellar and equiaxed microstructures of TC18 by adopting different heat treatment methods, and their research indicated that TC18 had better creep resistance [11]. Therefore, conducting research on the impact of the microstructure of high-temperature titanium alloys before and after heat treatment on creep life and creep mechanisms is essential.

In recent years, the influence of microstructure on the creep properties of high-temperature titanium alloys has aroused extensive research interest [11,12,13,14,15,16], and the creep of two-phase titanium alloys was mainly attributed to the slip of individual phases as well as the slip across phase boundaries [17]. It has been reported that a lamellar microstructure has better creep resistance than an equiaxed microstructure [11,18], and the creep resistance of basket-weave microstructure is superior to that of biphasic microstructure [14,15]. This has prompted many researchers to elucidate the relationship between microstructure and creep properties, proposing many typical creep mechanisms, such as dislocation gliding [8,11,15], grain boundary slip [19,20,21], twinning [1,22,23], and the precipitation of silicides [1,3,7]. Gu et al. indicated that the creep of TC11 at 450–550 °C was influenced by dislocation movement [15], and Zhang et al. showed that the creep mechanism of Ti65 in the transverse direction was grain boundary slip [20]. It is of vital importance to study the influence of the microstructure of different titanium alloys on creep properties and the corresponding creep mechanisms.

The TC25 alloy (the nominal composition is Ti-6.5Al-2Mo-1Zr-1Sn-1W-0.2Si) is a thermal-strength (α + β) biphasic titanium developed by the former Soviet Union in 1971 [24,25,26]. And due to the addition of the β-eutectoid element W and neutral element Sn, it has exhibited excellent mechanical properties at service temperature (at least 550 °C) [25,26]. He et al. [24] investigated the microstructure evolution of TC25 following deformation and heat treatment and indicated that the volume fraction of the globularized α phase increases with increasing deformation, and the amount of Mo in α phase decreases with globularization. Zhang et al. [27] studied the fatigue life of equiaxed, bimodal, and lamellar microstructures of TC25 and demonstrated that equiaxed and bimodal microstructures exhibited greater high-cycle fatigue resistance than the lamellar microstructure. Little research, however, has focused on the effects of heat treatment on the creep properties and creep mechanisms of TC25.

In this study, the microstructure change, tensile creep properties, and creep mechanism of TC25 before and after heat treatment at 550 °C were systematically studied. This work helps to comprehend the effect of heat treatment on the microstructure of TC25 titanium alloys, as well as the influence of microstructure on the tensile creep life and mechanism of TC25 titanium alloys. It has a certain guiding significance for the application of TC25 titanium alloys under high-temperature conditions.

## 2. Materials and Methods

### 2.1. Materials

The experimental materials used in this study were purchased commercially (Shenyang Enyiyou Technology Co., Ltd., Shenyang, China) and produced through vacuum arc melting, forging, and rolling. The chemical composition is listed in Table 1. The obtained materials were divided into two groups. One group remained in the original state (hereinafter referred to as TC25-1), and the other group was further subjected to solution treatment and aging treatment (hereinafter referred to as TC25-2). The (α + β)/β phase transition temperature (Tβ) of this alloy was 1018 °C [20,27], determined by high-temperature differential thermal analyzer. The heat treatment procedure for TC25-2 was solution treatment at 960 °C (below T_β_) for 2 h, followed by air-cooling to room temperature, and then aging at 550 °C for 3 h, followed by air-cooling to room temperature. Argon gas was used as the protective gas during the solution treatment and aging processes.

### 2.2. Experimental Procedures

**Figure 1:**
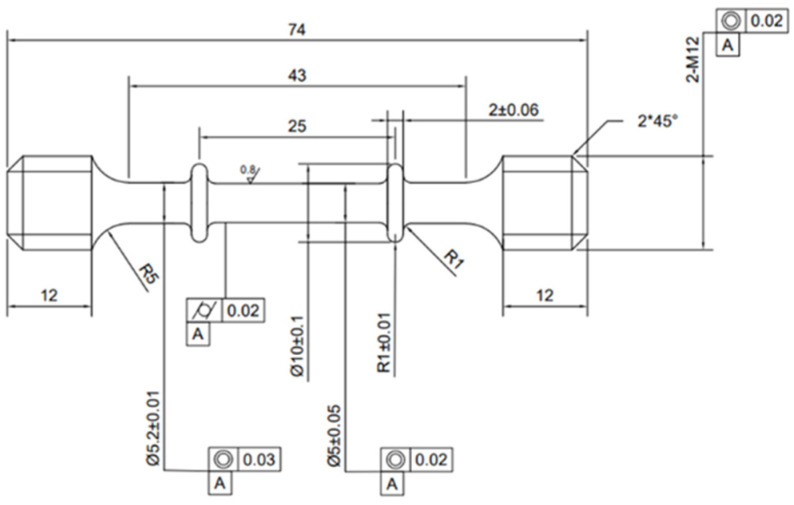
Geometric shape and dimensions of the tensile creep specimens (in mm).

In order to investigate the influence of microstructure on the tensile creep behavior of TC25-1 and TC25-2, wire cutting was performed on the two groups of materials. Figure 1 shows the processing details of the cylindrical specimen, with a gage length of 25 mm and a diameter of 5 mm [27]. The tensile creep tests were carried out on an electronic high-temperature creep testing machine (TSC105B, WANCE, Shenzhen, China). During the entire testing process, the whole specimen was kept at a constant temperature of 550 ± 2 °C in an electric resistance heating furnace [11]. Three kinds of stress, 500 MPa, 550 MPa, and 610 MPa, were applied in this study, and a prestress of 190 N was applied at each creep heating-up and heat preservation stage. In the electric resistance heating furnace, the specimen was heated to the designed test temperature with a heating rate of 5 °C/min and held for 1 h to stabilize the temperature over the gage section. The displacement and time data were collected during the testing process using the TestPilot_HTC_D01C version 1.0.1106 data collection and control software. All tests were not stopped until the specimen ruptured.

### 2.3. Microstructure Characterization

In order to reveal the effect of heat treatment on the TC25 alloy microstructure and the creep mechanism at 550 °C, various observational experiments were conducted. X-ray diffraction (XRD, Rigaku SmartLab 9Kw, Tokyo, Japan) equipped with Cu-Kα was used to identify the phase composition, with a scanning speed of 10°/min. An optical microscope (OM, Olympus GX71, Tokyo, Japan) and a scanning electron microscope (SEM, JEOL JSM-7800F, Tokyo, Japan) were used to observe the microstructure of the materials. The working voltage of the SEM was 15 KV, and the LDF mode was adopted. An energy-dispersive spectrometer (EDS, OXFORD XMAX 20, Britain, UK) was used to determine the element distribution. Meanwhile, backscatter diffraction was used to analyze the local orientation difference (EBSD, SYMMETRY S2, Britain, UK). The specimens for OM and SEM characterization were polished with a series of abrasive papers (#400-3000) and then chemically etched in corrosive liquid with a volume ratio of HF/HNO_3_/H_2_O = 1:3:7 [28]. The chemical etching time is about 1~2 s. After the etching, the specimens were ultrasonically cleaned in alcohol for 5 min. The EBSD specimens were polished with a series of abrasive papers and then thinned by argon ion, while the creep fracture surface was cleaned with alcohol and observed by SEM.

## 3. Results and Discussion

### 3.1. Initial Microstructure

**Figure 2:**
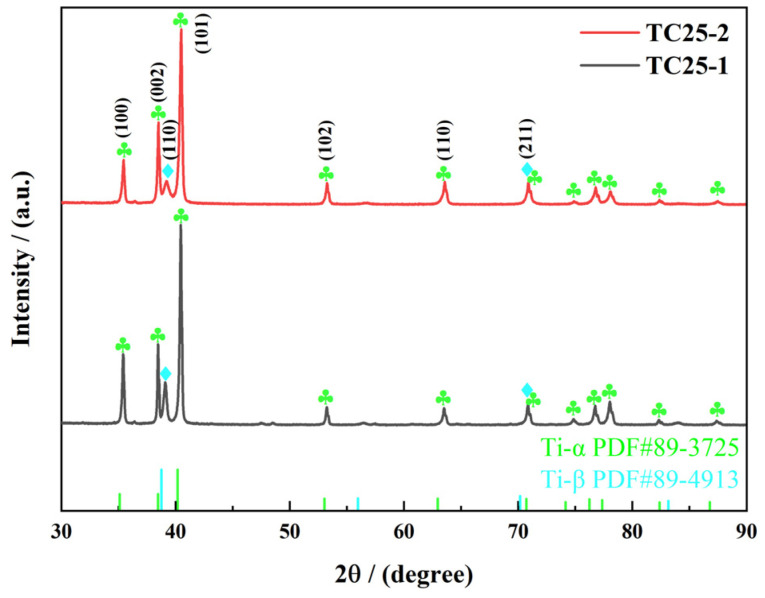
XRD patterns of TC25-1 and TC25-2 (the square represents Ti-α, and the club represents Ti-β).

The XRD results of TC25 before and after heat treatment are presented in Figure 2. It can be seen from Figure 2 that the main diffraction peaks of TC25 before and after heat treatment corresponded to α-titanium and β-titanium, which indicates that TC25 is mainly composed of the α and β phases [27]. The integral intensity of diffraction peaks is proportional to the volume fraction of the corresponding phase [29,30]. The relative intensity of the (110) diffraction peak (β-titanium) in TC25-2 is significantly lower than that in TC25-1, indicating that there is a transformation from the β phase to α phase during the heat treatment process [31,32].

**Figure 3:**
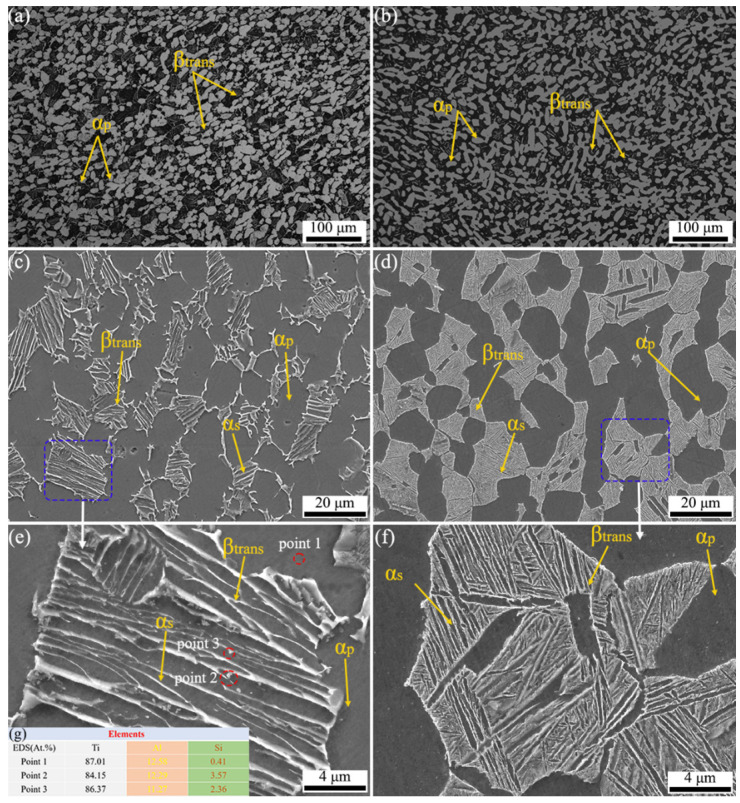
The microstructure of TC25-1 and TC25-2: (**a**,**b**) OM microstructure; (**c**,**d**) SEM microstructure; (**e**,**f**) regional amplification diagram (the blue dash line boxes in (**c**,**d**)); (**g**) elemental mapping in the red area of (**e**).

It has been reported that titanium alloys (TC25) which are used at high temperatures have bimodal structures composed of equiaxed primary α phase (αp) and transformed β matrix (βtrans); in particular, the β matrix (βtrans) consists of β ligaments and secondary α laths (αs) [33]. Figure 3a,b show the OM microstructures of TC25-1 and TC25-2; both TC25-1 and TC25-2 exhibit a typical bimodal microstructure in terms of α_p_ and β_trans_ phases. The heat treatment process made the adjacent α_p_ in TC25-1 grow to a larger α_p_ with a long elliptical shape in TC25-2. Image pro plus 6.0 was used to quantitatively analyze the content of α_p_ before and after heat treatment. The percentage content of α_p_ in TC25-1 is 51.8%, and that in TC25-2 is 55.7%. The increase in α_p_ content after heat treatment indicated that there was a transformation from the β_trans_ to α_p_ during the heat treatment process, which further confirmed the results of XRD patterns in Figure 2. Figure 3c,d show the enlarged TC25-1 and TC25-2 microstructure, respectively. It can be clearly observed that β_trans_ is filled with a large amount of α_s_ by bright contrast. After heat treatment, in TC25-2, α_s_ became thinner, presenting a needle-like microstructure, and arranged in parallel in multiple directions. The patterns of the α_s_ phase could be observed clearly after local magnification as Figure 3e,f showed, and the transformation from β_trans_ to α_p_ during the heat treatment process could also be obtained. In addition, small spherical precipitates were found in TC25-1 (the red dashed circle in Figure 3e), while no obvious precipitates were found after heat treatment, and the precipitates are mainly silicides [34,35,36], as the mapping results show in Figure 3g.

### 3.2. Tensile Creep Behavior

In order to evaluate the creep resistance of the alloy, creep tests were conducted at 550 °C under three different stresses (500 MPa, 550 MPa, 610 MPa). Creep is a temperature-sensitive thermal activation process that is generated by the combination of dislocation motion, atomic thermal motion, and stress [11,37]. The creep process is generally divided into three stages. Firstly, in the decelerating creep stage, plastic deformation and work hardening occurred in the alloy. Secondly, the steady-state creep stage is mainly caused by the cooperation of dislocations and obstacles, as well as the dislocation climb and slip [38]. Finally, in the third stage (accelerating creep stage), the creep rate increases sharply, and the creep damage continues to accumulate until the material fractures. Usually, the steady-state creep rate and creep rupture life are used to evaluate the creep resistance of materials [3,39].

**Figure 4:**
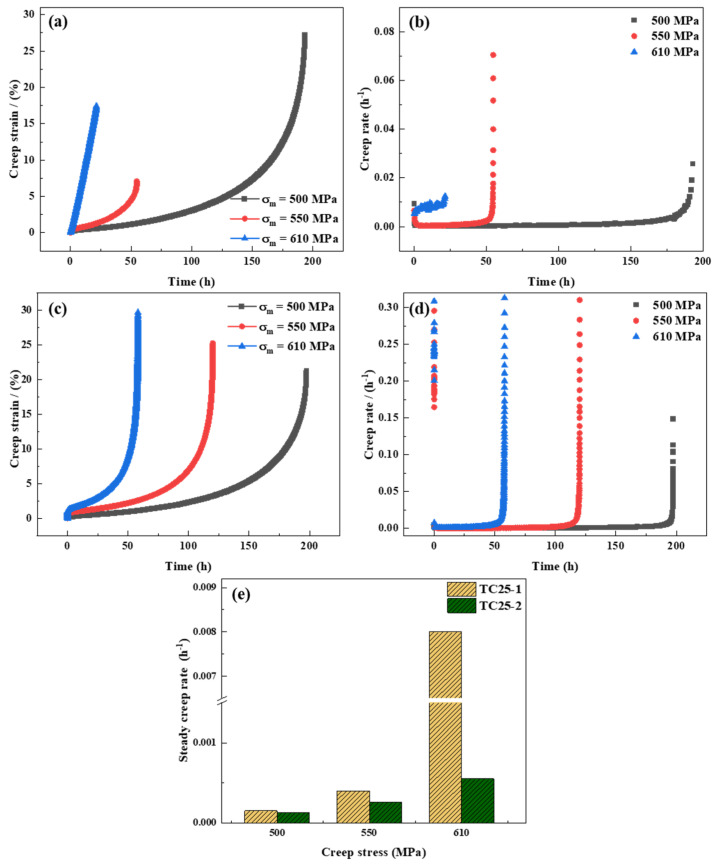
The creep properties of TC25-1 (**a**,**b**) and TC25-2 (**c**,**d**) at 550 °C: (**a**,**c**) creep curves; (**b**,**d**) creep rate curves; (**e**) steady-state creep rate.

Figure 4 displays the creep properties of TC25-1 and TC25-2. As Figure 4a shows, the curve indicates that under 610 MPa, the TC25-1 alloy had no obvious decelerating creep and steady-state creep stages, only an obvious accelerating creep stage. However, after heat treatment, there are obvious three creep stages, as Figure 4c shows. The steady-state creep stage accounts for approximately 20% (10 h) of the entire creep stage. Otherwise, it could be concluded that the TC25-1 remains with the accelerated creep stage as the main stage, and the steady-state creep stage emerges at 550 MPa and 500 MPa. The steady-state creep stage (550 MPa and 500 MPa) occupied 4% (2 h) and 12.5% (25 h) of the entire creep stage, respectively. For TC25-2, the steady-state creep stage accounts for 17% (20 h) and 18% (35 h) of the entire creep stage at 550 MPa and 500 MPa, respectively. In addition, under the high-stress conditions (610 MPa, 550 MPa), the creep life of TC25-2 (58 h, 128 h) exceeds twice that of TC25-1 (21 h, 54 h), as Figure 4a,c showed. And under the low-stress (500 MPa) condition, the creep life of TC25-2 (197 h) is still higher than that of TC25-1 (173 h). This indicates that the property of TC25 after heat treatment exhibits better creep resistance, as predicted. At the same temperature, the creep stress is an important parameter affecting the creep life [40]. Figure 4c indicates that the greater creep stress results in a shorter creep life and greater creep strain [11,41]. However, this phenomenon was not observed in Figure 4a, which may be caused by the microstructure and creep mechanism of titanium alloys [42].

The creep rate curves of TC25-1 and TC25-2 are presented in Figure 4b,d. It indicated that the steady-state creep rate increased with the increase in creep stress, whether before or after heat treatment. It could be found that under the same stress conditions (500 MPa, 550 MPa, 610 MPa), the steady-state creep rate of TC25-2 (3.6 × 10^−8^ s^−1^, 7.2 × 10^−8^ s^−1^, 1.5 × 10^−7^ s^−1^) was lower than that of TC25-1 (4.2 × 10^−8^ s^−1^, 1.1 × 10^−7^ s^−1^, 2.2 × 10^−6^ s^−1^). This phenomenon is more obvious in Figure 4e, allowing the conclusion that with the decrease in creep rate, the creep life increases. The creep mechanisms before and after heat treatment will be further discussed in Section 3.4.

### 3.3. Creep Fracture Mode

**Figure 5:**
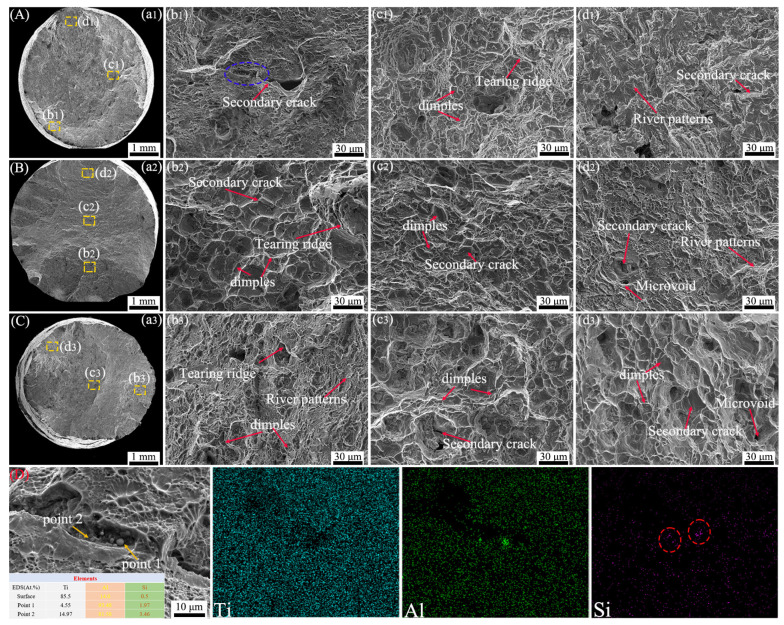
SEM creep fracture morphology of TC25-1 after creep tests (**A**) 500 MPa, (**B**) 550 MPa, (**C**) 610 MPa: (**a1**–**a3**) macroscopic fracture morphology; (**b1**–**d1**), (**b2**–**d2**), (**b3**–**d3**) morphological characteristics of typical microregions; (**D**) elemental mapping in the blue area of (**b1**).

Figure 5 shows the SEM morphology of TC25-1 after the creep test under different stresses at 550 °C. The macroscopic fractures (Figure 5(a1–a3)) indicate that no obvious necking behavior occurred under different stresses. Meanwhile, the shear lip regions similar to tensile fractures can be observed [28]. The microstructure in typical microregions of each group (Figure 5(b1–d1,b2–d2,b3–d3)) indicate that under three different stress conditions, the alloy exhibits a typical ductile fracture, with a large number of dimples of different sizes distributed [39]. Equiaxed α*_p_* tends to form dimples at the grain boundaries and transform into micropores [43]. Under the stress of 500 MPa, tearing ridges appeared at the edge of the dimples, indicating good toughness and plasticity of TC25-1 at this stress level. With the increase in stress, the size of tearing ridges became smaller and less obvious [42]. The appearance of the cleavage facets is usually considered as cleavage fracture, which is a characteristic of brittle fracture [42,44]. River patterns are found under different stress conditions, especially at 500 MPa (Figure 5(d1)), indicating that brittle fracture occurred during the creep fracture process. Figure 5D shows the element distribution of the blue area in Figure 5(b1), indicating that the spheroidized silicide grew further during the low-stress creep process. The Al element was often used as a stabilizing element for the α phase [26,45] while the Si element was often used as a stabilizing element for the β phase [15]. During the long-term creep process, the Si element often dynamically precipitated in the form of silicide, while the precipitation of the Al element was relatively less [46,47]. The contents of Al and Si elements in the spheroidized and grown silicide are much higher than those in the matrix, indicating that during the low-stress creep process, Al and Si elements diffused from the α_p_ to the spheroidized silicide. The creep life of TC25-1 at 500 MPa is much higher than that at 550 and 610 MPa (Figure 4a), indicating the silicon element mainly dynamically precipitated in the form of silicide and interacted with dislocations to improve the creep strength [46]. These results reveal that the creep fracture of TC25-1 is a mode combining brittle fracture and ductile fracture. Under low stress, element segregation is more likely to occur, and the characteristics of brittle fracture are more obvious.

**Figure 6:**
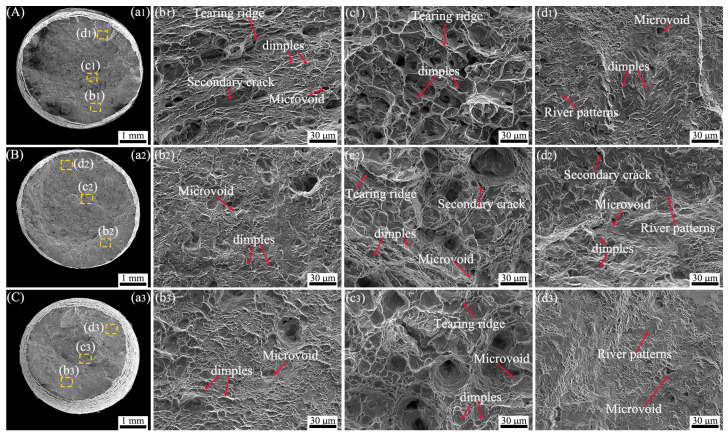
SEM creep fracture morphology of TC25-2 after creep tests at (**A**) 500 MPa, (**B**) 550 MPa, (**C**) 610 MPa: (**a1**–**a3**) macroscopic fracture morphology; (**b1**–**d1**), (**b2**–**d2**), (**b3**–**d3**) morphological characteristics of typical microregions.

Figure 6 shows the creep fracture surfaces of TC25-2 under different stresses at 550 °C. Figure 6(a1–c1) indicate that necking behavior occurs under different stress conditions, and the necking phenomenon becomes more pronounced with increasing stress compared with the TC25-1. This is consistent with the elongation of the specimen in the creep curves (Figure 4c). Overall, the creep fracture mode of TC25-2 is the same as that of TC25-1, which is a ductile fracture with micropore aggregation, accompanied by a large number of ductile dimples with different sizes. By comparing the tearing ridges near the dimples of the creep fracture surfaces of TC25-1 and TC25-2, it can be found that the tearing ridges of TC25-2 are more obvious, indicating that TC25-2 had a larger strain during the creep resistance process. In addition, around the dimples of TC25-2, there are a large number of microvoids, which are more than that of TC25-1, indicating that during the creep process, TC25-2 can disperse the stress, delay the crack initiation, and then have better creep resistance. In typical microscopic regions (Figure 6(b1,b2,c2), secondary cracks can be found. The secondary cracks after heat treatment are relatively fewer and shorter, indicating that it is difficult for cracks to initiate and propagate in the TC25-2 specimens. TC25-2 has smaller α_s_, which can prevent the transfer of cracks between different phases, thus having better creep resistance [48]. This was similar to basket-weave structures having better creep resistance [11]. In the microscopic regions of the creep fracture surfaces under different stresses (Figure 6(d1–d3)), the same river patterns as TC25-1 can be observed, indicating that brittle fracture is the fracture mode. However, the stress condition with obvious brittle fracture characteristics is 550 MPa, instead of 500 MPa for TC25-1. After heat treatment, the α/β interface of TC25-2 became smooth, the element distribution was more uniform, and no spheroidized β phase is found during the creep process. The above results show that the TC25-2 fracture mode is a mixed mode of ductile fracture and brittle fracture, with ductile fracture as predominating, and the refined α_s_ phase exhibits better creep resistance.

### 3.4. Creep Mechanism

According to the creep curves and creep performance results of TC25-1 and TC25-2, the steady-state creep rate is affected by the applied stress. The Arrhenius power law creep equation was used to evaluate the relationship between the steady-state creep rate and stress [15,49,50].

(1) $${\dot{\varepsilon }}_{s}=A\sigma^{n}\exp\left(-\frac{Q_{s}}{RT}\right)$$

where ${\dot{\varepsilon }}_{s}$ is steady-state creep rate (s^−1^), A is the material constant, σ is flow stress (MPa), *n* is the apparent stress index, $Q_{s}$ is creep apparent activation energy (KJ/mol), R is the gas constant (8.314 J/(mol K)), and T is Kelvin temperature (K). The deformation mechanism of the creep process can be expressed by the apparent stress index n. Equation (1) is applicable to the case where the apparent activation energy is equal to the activation energy of metal self-diffusion [15]. According to Equation (1), the expression of n can be derived as Equation (2), as follows:

(2) $$n={\left(\frac{\partial ln\dot{\varepsilon }}{\partial ln\sigma }\right)}_{T}$$

**Figure 7:**
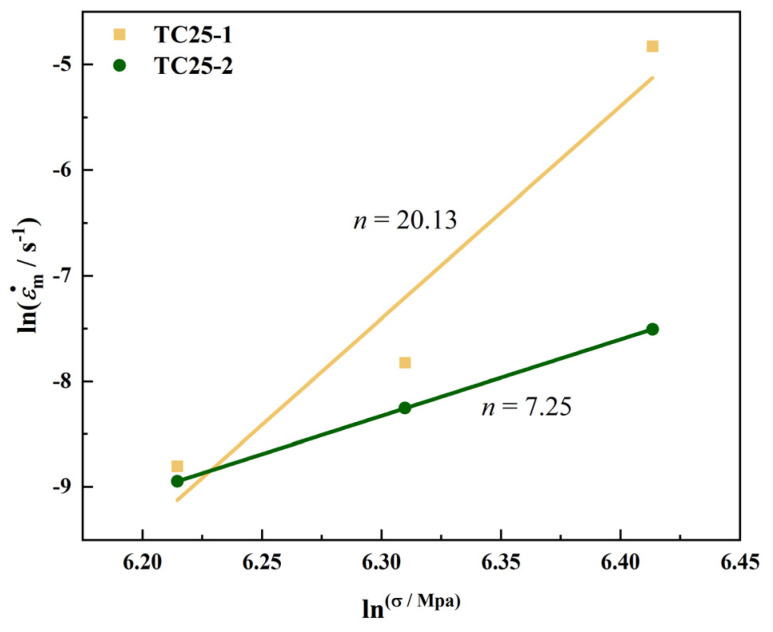
The relationship of steady-state creep rate and logarithm of stress.

Figure 7 shows the curves of the natural logarithmic creep steady-state rate versus stress of TC25-1 and TC25-2 at 550 °C. Usually, *n* = 1 indicates that the creep process is controlled by the diffusion of vacancies along the grain boundaries [51]; *n* = 2–3 indicates that the creep process is controlled by the diffusion of vacancies and dislocation climb [31,50]; *n* = 4–8 indicates that the creep process is controlled by dislocation climb [52]. As shown in Figure 7, the apparent stress index *n* of TC25-1 and TC25-2 is calculated to be 20.13 and 7.25, respectively, by using OriginPro 2024 software. It indicates that the creep mechanism of TC25-2 is dislocation climb. Dislocations may slide within the α_p_ and accumulate at the α_p_/β_trans_ grain boundaries. However, the apparent stress index *n* of TC25-1 is much greater than 8, indicating that there are factors hindering creep in the material before heat treatment near 550 °C [42]. By comparing the apparent stress index of TC25-1 and TC25-2, it could be found that the n value before heat treatment is much greater than that after heat treatment, indicating that TC25-1 is more sensitive to creep stress. The creep mechanisms before and after heat treatment are further analyzed below through the microstructure near the creep fracture and EBSD.

**Figure 8:**
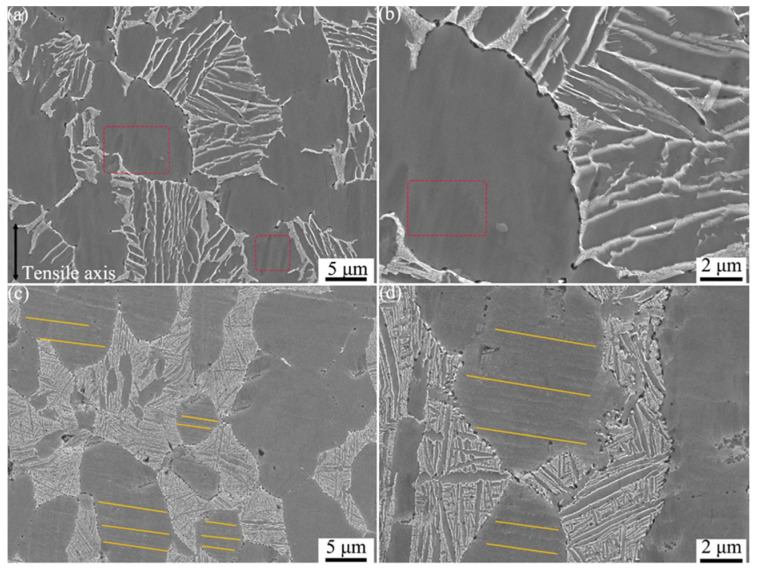
SEM images of the regions near the creep fracture at 550 °C/550 MPa: (**a**,**b**) TC25-1, (**c**,**d**) TC25-2.

The scanning electron microscope secondary electron (SE) images of the microstructures near creep fracture regions of TC25-1 and TC25-2 are shown in Figure 8. The α_p_ of TC25-1 after creep test presents obvious tensile traces in the stress loading direction. Its main deformation region is circled with a red square in Figure 8a. The tensile traces in Figure 8b are more obvious, indicating that lattice torsion occurred in the α phase during the creep process. In addition, obvious cracking occurs at the grain boundary of α_p_/β_trans_, indicating that during the creep process, the fracture first starts from the grain boundary, and the creep mechanism is grain boundary sliding. It has been found in many studies that grain boundary sliding is a common creep mechanism in titanium alloys [17,21]. The larger microcracks formed at the convex interface of α_p/_β_trans_ could be found, and they gradually expand to both sides, eventually causing grain boundary cracking, forming large cracks, and finally leading to creep fracture. This is consistent with the large cracks observed in the creep fracture surface (Figure 5).

Compared with TC25-1, no obvious tensile traces are observed in TC25-2, indicating that the creep mechanism changed after heat treatment. On the contrary, many slip lines perpendicular to the grain boundary of α_p_/β_trans_ are found in Figure 8c, indicating that a large number of dislocations occurred during the creep process of TC25-2. The dislocations slipped along the α_p_ matrix, forming parallel slip lines [53,54]. The slip lines of the substrate are more obvious in Figure 8d, indicating that the creep mechanism of TC25-2 is dislocation climb, which is consistent with the derivation result of the Arrhenius power law creep equation. It is worth noting that no obvious slip lines are observed in the large α_p_ phase, which is caused by the Burger vector being parallel to the sample surface and the underdeveloped slip bands. It can be observed from Figure 8c that the slip lines originated from the interior of the α_p_ phase rather than the α_p_/β_trans_ interface, and the slip lines terminated at the α_p_/β_trans_ interface without crossing the grain boundary. Compared with the microstructure of TC25-2 before creep (Figure 3f), obvious fragmentation occurred at the α_p_/β_trans_ grain boundary. Cracks formed between the interfaces of the fragmented α_p_ and β ligaments and did not exhibit aggregative behavior. This indicates that during the creep process, in order to adapt to the large creep stress and avoid stress concentration, the β ligaments are fragmented, thus resulting in better creep performance. The fragmented β ligaments would further form large dimples during the creep process (Figure 6), which is not found in TC25-1. This indicates that the structure of the grain boundary has a significant influence on the creep performance [55]. In this study, the smooth grain boundary structure has better creep performance. The refined α_s_ interface of TC25-1 also presents tiny pores (Figure 8d), indicating that the α_s_ prevents the concentration of stress and enhances the creep resistance during the creep process, which is not found in TC25-1.

**Figure 9:**
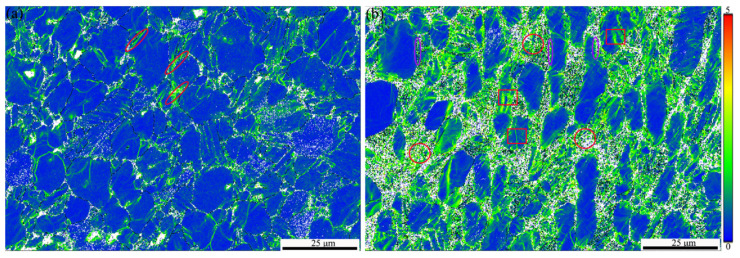
Kernel average misorientation (KAM) analysis near the creep fracture at 550 °C/550 MPa: (**a**) TC25-1, (**b**) TC25-2.

Kernel average misorientation (KAM) maps can be used to characterize the stress distribution and geometric dislocation density distribution of the specimen during the deformation process [20,56]. Therefore, in order to further analyze the creep mechanisms of TC25 before and after heat treatment, KAM maps were used to characterize the dislocation distribution to a certain extent. Figure 9 shows the dislocation distribution in the vicinity after the creep fracture of TC25-1 and TC25-2. The area with a higher KAM value indicates higher stress and a greater geometric necessary dislocation density. In Figure 9a, the stress concentration area is mainly at the grain boundaries of α_p_/β_trans_, indicating that dislocations mainly accumulate at the grain boundaries of α_p_/β_trans_. This is prone to cause grain boundary sliding and further leads to grain boundary cracking, which is consistent with the phenomenon observed in Figure 8a. In addition, the red elliptical area in Figure 9a indicates that dislocations present a continuous banded distribution at grain boundaries of α_p_/β_trans_, thereby causing cracks to propagate along the interface in TC25-1 (Figure 8b). On the contrary, in Figure 9b, the stress concentration area appeared not only at the grain boundaries of α_p_/β_trans_ but also in α_s_ (red circle), indicating that the stress distribution during the creep process of TC25-2 is relatively uniform and could well improve the creep resistance. Furthermore, the fragmentation occurs in the β ligaments with a point-like dislocation distribution in the fragmented area (purple elliptical circle), which indicates that the β ligaments fragmented to further improve the creep resistance in order to adapt to the high-stress condition during the creep process. The stress concentration behavior can also be obviously observed in the small α_p_ (red square frame), which is consistent with the phenomenon in Figure 8c. The above results indicate that the creep mechanism of TC25 is grain boundary sliding before heat treatment and dislocation climb after heat treatment, and the improvement of creep performance after heat treatment mainly originated from the smooth α_p/_β_trans_ grain boundary and the refined α_s_.

## 4. Conclusions

In summary, the microstructure changes, creep properties, and creep mechanism of the TC25 high-temperature alloy before and after heat treatment were studied. The TC25 high-temperature alloy exhibited a typical bimodal microstructure. After solution treatment (960 °C/2 h/AC) and aging treatment (550 °C/3 h/AC), the α_p_/β_trans_ grain boundaries of TC25 became smoother, and the αs became finer. Heat treatment greatly improved the creep resistance of TC25, especially under high- and medium-stress conditions. Its creep life increased by approximately twice compared to that before heat treatment. At the same temperature (550 °C), the increase in stress would accelerate the creep process, resulting in a decrease in creep life and an increase in the steady-state creep rate. The main reason for the enhancement of creep resistance after heat treatment was attributed to the fact that the smooth α_p_/β_trans_ grain boundaries broke during the creep process, dispersed the creep stress, and prevented dislocation climb, and the refined α_s_ could effectively prevent the dislocation movement. The creep fracture modes of TC25 before and after heat treatment are both mixed fractures combining ductile fracture and brittle fracture. The creep mechanism before heat treatment is grain boundary sliding, and the creep mechanism changed to dislocation climb after heat treatment.

## Figures and Tables

**Table 1:** Chemical composition of TC25 alloy (wt. %).

Ti	Al	W	Zr	Mo	Sn	Si
Bal.	6.48	0.72	1.02	1.78	1.12	0.13

## Data Availability

The raw/processed data required to reproduce these findings cannot be shared at this time as the data also forms part of an ongoing study.
